# Growth profile of *Carboxydothermus hydrogenoformans* on pyruvate

**DOI:** 10.1186/2191-0855-3-60

**Published:** 2013-10-07

**Authors:** Mathieu Haddad, Ruxandra Cimpoia, Ya Zhao, Serge R Guiot

**Affiliations:** 1 Department of Microbiology, Infectiology and Immunology, Université de Montréal, Montreal H3C 3J7, Canada; 2 Bioengineering Laboratory, Energy, Mining and Environment, National Research Council Canada, 6100 Royalmount Avenue, Montreal H4P 2R2, Canada; 3 Institute of Biophysics, Chinese Academy of Sciences, Chaoyang District, Beijing 100101, P.R. China

**Keywords:** Carbon monoxide, Water-gas shift reaction, *C. hydrogenoformans*, Pyruvate

## Abstract

*Carboxydothermus hydrogenoformans* is a thermophilic anaerobic strain most widely known for its ability to produce hydrogen (H_2_) when grown on carbon monoxide (CO). Although relatively well studied, growth characterization on pyruvate has never been assessed. The present work fully characterizes growth of the bacterium on pyruvate as a sole carbon source. *C. hydrogenoformans* demonstrated a growth rate of 0.03 h^-1^, with pyruvate consumption ranging between 0.21 and 0.48 mol · g^-1^ volatile suspended solid · d^-1^. A lag phase was also observed when switching from pyruvate to CO. When grown simultaneously on pyruvate and CO, pyruvate consumption was initiated upon CO depletion. This was attributed to pyruvate oxidation inhibition by CO, and not to a diauxic phenomenom. The strain also showed homoacetogenic activity.

## Introduction

Gasification of biomass and waste is a way to produce non-fossil hydrogen and involves a high temperature (750–1500°C) conversion of any carbonaceous material into a synthesis gas (syngas) mainly composed of carbon monoxide (CO), carbon dioxide (CO_2_) and hydrogen (H_2_) (Rezaiyan and Cheremisinoff 2005Song 2010). The water-gas shift (WGS) reaction widely utilized in industry can be used to augment the H_2_ content of syngas. In this process, steam reacts with CO to produce carbon dioxide and hydrogen (CO + H_2_O → CO_2_ + H_2_).

Despite its deleterious effects on many living species, carbon monoxide is also the starting point for many food chains, especially in hydrothermal environments such as the deep sea, hot springs, and volcanoes. *Carboxydothermus hydrogenoformans* is one such organism that was isolated from a hot spring on Kunashir Island, Russia (Svetlichnyi et al. 1991). This extreme thermophilic bacillus uses CO as sole source of carbon and energy while catalyzing the WGS reaction.

Gasifiers, as all industrial machinery, may need to be stopped for maintenance or for emergency intervention. Considering the high decay rate of *C. hydrogenoformans* (Zhao et al. 2013), it is mandatory to have a secondary carbon and energy source that would act as a back-up system in case CO fails to be delivered over an extended period of time to the biomass. That way, biomass concentration can be maintained, allowing a smooth restart of the WGS reaction with no lag time or the need to rebuild the bacterial population. In this regard, pyruvate could prove to be an interesting substitution substrate since *C. hydrogenoformans* was reported to be able to grow on it as an alternative carbon and electron source to carbon monoxide (Svetlichnyi et al. 1994). Pyruvate fermentation in anaerobic fermentative organisms leads to the production of acetyl-CoA and hydrogen (Hallenbeck and Benemann 2002). This reaction is catalyzed by two enzymes (Thauer et al. 1972Carere et al. 2008): the pyruvate:ferredoxin oxidoreductase (POR), which catalyzes: pyruvate + CoA + 2 Fd_ox_ → acetyl-CoA + CO_2_ + Fd_red_ + 2H^+^; and the pyruvate: formate lyase (PFL), which catalyzes: pyruvate + CoA → acetyl-CoA + formate.

As the reported growth information of *C. hydrogenoformans* on pyruvate was only qualitative, the objective of this study was to quantitatively assess its growth and activity kinetics on pyruvate and elucidate a potential impact of substrate alternation on both activities and products formation.

## Materials and methods

### Culture medium

*C. hydrogenoformans* (DSM 6008) was obtained from the German Collection of Microorganisms and Cell Cultures (DSMZ, Braunschweig, Germany). The strain was cultivated in shake-culture at 100 rpm under strictly anaerobic conditions at 70°C in a basal mineral medium buffered with a bicarbonate-phosphate buffer. The medium as formulated by Zhao et al. (2011) contained (in g · L^–1^ of demineralized water): KCl 0.33, MgCl_2_ · 6H_2_O 0.102, CaCl_2_ · 2H_2_O 0.015, NH_4_Cl 0.33, KH_2_PO_4_ 0.136, NaHCO_3_ 0.42, yeast extract 0.05, Na_2_S · 9H_2_O 0.7. The medium was supplemented with 10 mL · L^–1^ trace metals solution and 10 ml · L^–1^ of vitamins solution prepared as described previously (Stams et al. 1993). All stock solutions were autoclaved, except for the vitamin solution, which was sterilized by filtration through 0.22 μm filter membranes. The initial pH was adjusted between 6.8 and 7.0.

### Growth quantification

The pyruvate activity tests were conducted in 120 mL serum bottles in quintuplicate. Bottles were filled with 30 mL of the growth medium, inoculated with 2 mL of *C. hydrogenoformans* at an initial concentration of 6.65 mg volatile suspended solid (VSS) · L^–1^, capped, and flushed with a gas mixture of N_2_/CO_2_ to establish anaerobic conditions. Starter cultures were active *C. hydrogenoformans* cultures that were cultivated on CO as sole source of carbon and energy. Headspace monitoring of these cultures was done prior to inoculation to ensure that they were not in latency phase. Bottles were then flushed with a high purity CO gas (100%) and set to atmospheric pressure or spiked with a pyruvate solution to an initial concentration of 3.0 ± 0.1 g · L^–1^, and incubated in the absence of light at 70°C and 100 rpm in a rotary shaker (New Brunswick, Edison, NJ).

Microbial quantification in the bottles was performed immediately after substrate feeding (time 0), then intermittently until the end of experiment. Biomass quantification was achieved using chemical oxygen demand (COD) measurements which were then converted to VSS using a factor of 1.37 g COD · g^–1^ VSS based on the elemental formula of microbial biomass as CH_1.79_O_0.5_N_0.2_S_0.005_ (Roels 1983).

Both substrate (CO, pyruvate) depletion and catabolite (H_2_, CO_2_, volatile fatty acids (VFA) and alcohols) production were monitored. The specific substrate uptake or product formation rates, expressed as mol · g^–1^ VSS · d^–1^, were obtained by the rate of depletion or accumulation (mol · d^–1^) per bottle divided by the number of grams of biomass-VSS as estimated in the bottle. The hydrogen yield (Y_H2_) was expressed as a percentage of the H_2_ gas produced per CO consumed (mol/mol).

### Analytical methods

The COD was determined according to Standard Methods (Eaton et al. 2005), using a spectrophotometer DRB 200 (Hach Company, Loveland, CO).

Gas samples were obtained by withdrawing 300 μL of gas from the bottle headspace using a gas-tight syringe (model 1750 Hamilton, Reno, NV). Gas composition (H_2_, CO, CO_2_) was measured by injecting this gas into a HP 6890 gas chromatograph (Hewlett Packard, Palo Alto, CA) equipped with a thermal conductivity detector (TCD) and a 11 m × 3.2 mm 60/80 mesh Chromosorb 102 packed column (Supelco, Bellafonte, PA). The column temperature was held at 60°C for 7 min and increased to 225°C at a rate of 60°C per min. Argon was used as the carrier gas. The injector and detector were maintained at 125°C and 150°C respectively.

VFAs (i.e. acetic, propionic and butyric acids) were measured on an Agilent 6890 (Wilmington, DE) gas chromatograph (GC) equipped with a flame ionization detector (FID). 0.2 μL samples were diluted 1:1 (vol./vol.) with an internal standard of iso-butyric acid in 6% formic acid, directly injected on a glass column of 1 m × 2 mm Carbopack C (60–80 mesh) coated with 0.3% Carbowax 20M and 0.1% H_3_PO_4_. The column was held at 130°C for 4 min. Helium was the carrier gas, fed at a rate of 20 mL · min^–1^. Both injector and detector were maintained at 200°C.

For measurement of solvents (methanol, ethanol, acetone, 2-propanol, tert-butanol, n-propanol, sec-butanol, n-butanol) 100 μL of liquid was transferred into a vial that had 20 mL of headspace and was crimp sealed with a Teflon-coated septum. The vial was heated at 80°C for 2 min, then 1000 μL of headspace gas was injected onto a DB-ACL2 capillary column of 30 m × 530 mm × 2 μm using a Combipal autosampler (CTC Analytics AG, Zwingen, Switzerland). The column was held at 40°C for 10 min. Helium was the carrier gas at a head pressure of 5 psi. The injector and the detector were maintained at 200°C and 250°C, respectively.

Pyruvate was monitored using a high performance liquid chromatograph (HPLC) (Waters, Milford, MA) equipped with a model 600 pump, a model 717 Plus autosampler and a refractive index detector (model 2414) linked to a photodiode array (PDA) detector (model 2996). The separation was made on a 300 mm × 7.8 mm ICSep IC ION-300 column (Transgenomic, Omaha, NE). The mobile phase was 0.01 N H_2_SO_4_ at 0.4 mL · min^–1^. Analyses were carried out at 35°C.

## Results

**Figure 1:**
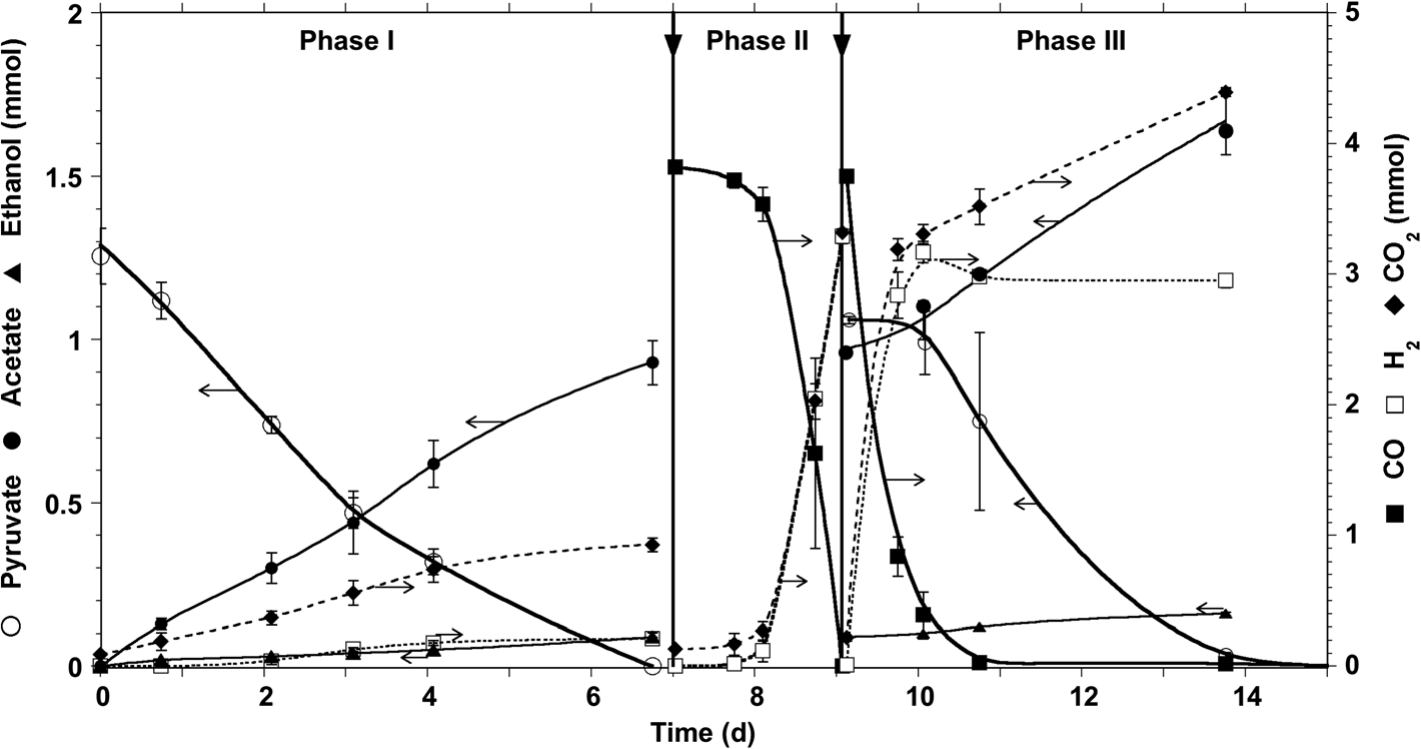
**Time-course of CO and pyruvate consumption and hydrogen CO**_**2**_**, acetate and ethanol production, by the *C. hydrogenoformans *culture when alternating substrate.** Phase I, Pyruvate only; Phase II, CO only; Phase III: CO and pyruvate; the vertical arrows indicate the change of substrate; horizontal arrows indicate the axis which the data refer to.

*C. hydrogenoformans* was reported to grow on pyruvate as an alternative carbon and electron source to carbon monoxide (Svetlichnyi et al. 1994). *C. hydrogenoformans* activity and formed products were first quantified on pyruvate (3.0 ± 0.1 g · L^-1^) with an initial microbial concentration of 6.65 mg VSS · L^-1^ for a feed-to-microorganisms ratio (F/M) of 515. (Figure 1, Phase I). Over phase I, the highest pyruvate consumption activity (initial rate reported on initial biomass concentration) and the lowest activity (final rate reported on final biomass concentration) were 0.48 ± 0.27 and 0.21 ± 0.03 mol · g VSS^-1^.d^-1^, respectively. The corresponding product yields were 77, 7, 13 and 75% (mol/mol pyruvate) for acetate, ethanol, hydrogen and CO_2_, respectively. The formation of trace methanol and 2-propanol were also observed. Once pyruvate was depleted, the cultures were flushed with CO (90 mL headspace filled with 100% CO, 1 bar) only (phase II, Figure 1), then with CO and pyruvate simultaneously (phase III, Figure 1), at the same concentrations used in phases I and II. Substrate consumption (CO and pyruvate) and products formation (H_2_, CO_2_, acetate, ethanol) were continuously monitored. The results showed that substrate alternation had neither an impact over products yield nor on CO or pyruvate consumption rates. The 17 hours lag time in pyruvate consumption at the beginning of phase III was expected due to the simultaneous addition of CO and pyruvate at the end of phase II. Pyruvate consumption did not begin before 90% of the CO was consumed, indicating clear sequential substrate consumption. A significant discrepancy of 0.72 mmol between CO_2_ and H_2_ content occurred when *C. hydrogenoformans* was grown on pyruvate in both phases I and III, clearly indicating that part of the H_2_ produced was also consumed. This difference could not be explained by the production of ethanol alone (0.16 mmol). Interestingly, hydrogen consumption stopped once ethanol concentration in the medium reached 5.33 mmol · L^-1^ (phase III).

**Figure 2:**
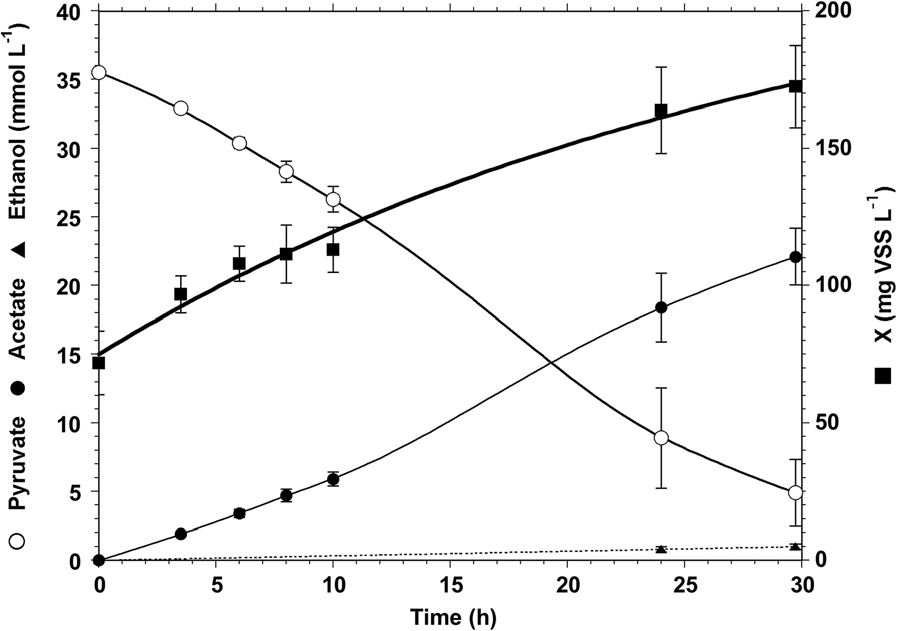
**Time courses of *C. hydrogenoformans *growth (X), pyruvate consumption, and acetate and ethanol production.**

Concurrently, an independent fresh growth experimental set was conducted on pyruvate alone to accurately assess the *C. hydrogenoformans* growth rate and yield on pyruvate, verify the reproducibility of the above results (Figure 2) and confirm the absence of lag time for growth and substrate consumption when CO is not present. The initial pyruvate concentration was 3.0 ± 0.1 g · L^-1^, with an initial microbial concentration of 71.9 mg VSS · L^-1^ for F/M of 43. Pyruvate consumption started within the first 4 hours compared to over 22 hours when in simultaneous presence of CO and pyruvate (Figure 1, Phase III). The observed growth rate was 0.03 ± 0.005 h^-1^. Pyruvate consumption activity was stable throughout the experiment with the highest and lowest activity of 0.24 and 0.21 mol · g VSS^-1^.d^-1^, respectively. This corresponded to apparent growth yields of 3.51 ± 0.69 g VSS · mol^-1^ pyruvate (i.e. 4.28 ± 0.54% by weight), respectively. The product yields were 72, 24 and 3% (mol/mol pyruvate) for acetate, hydrogen and ethanol, respectively.

## Discussion

Since pyruvate consumption by *C. hydrogenoformans* has not been thoroughly described in literature (Svetlichnyi et al. 1994), the present work allows a better understanding of this metabolism. When switching from CO to pyruvate, little lag time was observed versus the 17h lag observed for the inverse (pyruvate to CO). This could be explained by the number of steps involved in pyruvate degradation (Knappe and Sawers 1990Bock and Schonheit 1995) as compared to CO. Indeed, *C. hydrogenoformans* is able to convert pyruvate to acetyl-CoA in a single step, using its pyruvate-ferredoxin oxidoreductase (POR) (Perumal et al. 2012). On the other hand, CO transformation to acetyl-coA requires 4 steps and three enzymes (Lu et al. 1990): the bifunctional CODH/ACS complex, the monofunctional CODH, and a methylated corrinoid iron–sulfur protein (Ragsdale 2008). Also, the CO-related genes (coo operon) were found to be induced upon CO binding on the sensor and the transcriptional regulatory CooA protein (He et al. 1996Aono et al. 2000Wu et al. 2005). When assessing the activity profiles of the enzymes involved in pyruvate catabolism in *Clostridium thermocellum* (Rydzak et al. 2009), it was found that the expression level of the POR enzyme remained constant as growth progressed from early exponential to stationary phase. Hence, it was concluded that the changes of enzyme expression involved in pyruvate catabolism were negligible in response to growth (Rydzak et al. 2009). Assuming that the expression pattern of the POR superfamily is similar across *Clostridia*, these findings suggest that the POR enzyme is constantly expressed under anaerobic conditions as opposed to the CO-induced CODH (Sawers and Böck 1989Knappe and Sawers 1990).

When *C. hydrogenoformans* was simultaneously fed with pyruvate and CO, the substrates were sequentially consumed, the pyruvate being consumed only when CO is depleted. This has the appearance of a diauxie phenomenon, also named carbon catabolite repression (CCR) (Gorke and Stulke 2008), and defined as a regulatory phenomenon during which, in the presence of two carbon sources, gene expression and protein activity of the secondary carbon source are reduced. However in this case, the thermodynamics suggested that pyruvate should have been consumed before CO. The Gibbs free energy changes at 70°C and neutral pH were as follows: ∆G’ = - 45 kJ/mol for the the reaction CO + H_2_O → CO_2_ + H_2_, with p_CO_ at 1.1 atm, p_CO2_ at 0.06 atm and p_H2_ at 0.01 atm; ∆G’ = - 81 kJ/mol, for the reaction CH_3_COCOO^-^ + 2 H_2_O → CH_3_COO^-^ + HCO_3_^–^ + H^+^ + H_2_, with p_H2_ 0.01 atm, [CH_3_COCOO^-^] 0.04 M, [CH_3_COO^-^] 0.03 M, [HCO_3_^–^] 0.005 M. This thermodynamic prediction is consistent with the observed growth kinetics, with a faster rate for pyruvate (μ 0.03 h^-1^) than on CO (μ 0.01 h^-1^) (Zhao et al. 2013). Thus diauxie, *sensu stricto*, cannot be retained as an explanation. The substrate consumption sequence as observed, is probably due to CO toxicity to the pyruvate pathway present in *C. hydrogenoformans* (Uniprot accession numbers: Q3ADQ7; Q3AFU1; Q3AFU0; Q3ACZ5). CO has proven to be a potent inhibitor to pyruvate oxidation in another strictly anaerobic hyperthermophile, *Pyrococcus furiosus* (Blamey and Adams 1993) and inhibited the hydrogenase activity of the POR in *Clostridium thermoaceticum* (Menon and Ragsdale 1996).

The observed discrepancy between H_2_ and CO_2_ content during pyruvate fermentation (Figure 1) with no subsequent by-product formation besides acetate strongly suggests a homoacetogenic activity of the bacterial strain. All the genes in the acetyl-CoA pathway have been identified in the *C. hydrogenoformans* genome (Wu et al. 2005) making CO_2_ reduction to acetate possible (2 CO_2_ + 4 H_2_ → CH_3_COO^-^ + H^+^ + 2 H_2_O). The stoichiometry-based combination of this homoacetogenic reaction with the pyruvate-to-acetate and pyruvate-to-ethanol reactions predicts a CO_2_ net production which is nearly 4 times higher than that of hydrogen, when the yields observed for acetate, ethanol and hydrogen are used as actual stoichiometric coefficients of the balanced chemical equation: this is quite in line with the phase I observations. Adding to this, acetogenic shift of *C. hydrogenoformans* from CO was showed in final stages of incubation (Henstra and Stams 2011). Hence, homoacetogenesis would explain the linear increase in acetate in phase III (Figure 1) prior to pyruvate consumption.

In phase III (Figure 1), although hydrogen (issued from the WGS reaction) and pyruvate were present in large amounts, and that ethanol production from pyruvate and hydrogen (∆G°’ = -57 kJ/reaction) is more thermodynamically favourable than acetate production from pyruvate (∆G°’ = -47 kJ/reaction), hydrogen was not significantly consumed while ethanol did not reach a concentration higher than 5.3 mM. This could be explained by the fact that the alcohol dehydrogenase present in *C. hydrogenoformans* (NCBI Accession AF244667) is inhibited by ethanol as it was demonstrated in another thermophilic clostridium, *C. thermocellum*, at an even lower ethanol concentration (0.14 mM) (Lamed and Zeikus 1980Lovitt et al. 1988).

To conclude, the present work revealed new growth-related features of *C. hydrogenoformans.* Growth of the bacterium on pyruvate as sole C source was fully characterized. When grown simultaneously on pyruvate and CO, pyruvate consumption was initiated upon CO depletion. This was attributed to inhibition of pyruvate oxidation by CO. While the bulk of acetate derived from pyruvate, it is likely that there was also some homoacetogenic activity, thus contributing to the formation of acetate from H_2_ and CO_2_, which had never previously been shown with *C. hydrogenoformans*.

## Competing interests

The authors declare that they have no competing interests.

## Authors’ contributions

RC and YZ designed the experiment; YZ and MH carried out the experimental work; RC, MH and SRG interpreted the results; MH and SRG wrote the manuscript. All authors read and approved the final manuscript.
